# Protective Effects of PACAP in a Rat Model of Diabetic Neuropathy

**DOI:** 10.3390/ijms221910691

**Published:** 2021-10-02

**Authors:** Peter Kiss, Eszter Banki, Balazs Gaszner, Daniel Nagy, Zsuzsanna Helyes, Endre Pal, Gyongyver Reman, Gabor Toth, Andrea Tamas, Dora Reglodi

**Affiliations:** 1 Department of Anatomy, MTA-PTE PACAP Research Team, Centre for Neuroscience, Szentagothai Research Centre, University of Pecs Medical School, Szigeti ut 12, H-7624 Pecs, Hungary; peter.kiss@aok.pte.hu (P.K.); bankieszti@gmail.com (E.B.); balazs.b.gaszner@aok.pte.hu (B.G.); gy.reman@gmail.com (G.R.); andreatamassz@gmail.com (A.T.); 2 Department of Pharmacology and Pharmacotherapy, Szentagothai Research Centre, University of Pecs Medical School, Szigeti ut 12, H-7624 Pecs, Hungary; dnagy4@gmail.com (D.N.); zsuzsanna.helyes@aok.pte.hu (Z.H.); 3 Department of Neurology, University of Pecs Clinical Centre, Ret utca 2, H-7623 Pecs, Hungary; pal.endre@pte.hu; 4 Neuropathology Unit, Department of Pathology, University of Pecs Clinical Centre, Rakoczi ut 2, H-7623 Pecs, Hungary; 5 Department of Medical Chemistry, Faculty of Medicine, University of Szeged, Dom ter 8, H-6720 Szeged, Hungary; toth.gabor@med.u-szeged.hu

**Keywords:** diabetes, PACAP, neuroprotection, myelin, dorsal horn, periaqueductal grey

## Abstract

Pituitary adenylate cyclase-activating peptide (PACAP) is a neuropeptide with a widespread occurrence and diverse effects. PACAP has well-documented neuro- and cytoprotective effects, proven in numerous studies. Among others, PACAP is protective in models of diabetes-associated diseases, such as diabetic nephropathy and retinopathy. As the neuropeptide has strong neurotrophic and neuroprotective actions, we aimed at investigating the effects of PACAP in a rat model of streptozotocin-induced diabetic neuropathy, another common complication of diabetes. Rats were treated with PACAP1-38 every second day for 8 weeks starting simultaneously with the streptozotocin injection. Nerve fiber morphology was examined with electron microscopy, chronic neuronal activation in pain processing centers was studied with FosB immunohistochemistry, and functionality was assessed by determining the mechanical nociceptive threshold. PACAP treatment did not alter body weight or blood glucose levels during the 8-week observation period. However, PACAP attenuated the mechanical hyperalgesia, compared to vehicle-treated diabetic animals, and it markedly reduced the morphological signs characteristic for neuropathy: axon–myelin separation, mitochondrial fission, unmyelinated fiber atrophy, and basement membrane thickening of endoneurial vessels. Furthermore, PACAP attenuated the increase in FosB immunoreactivity in the dorsal spinal horn and periaqueductal grey matter. Our results show that PACAP is a promising therapeutic agent in diabetes-associated complications, including diabetic neuropathy.

## 1. Introduction

Pituitary adenylate cyclase-activating peptide (PACAP) is a member of the vasoactive intestinal peptide (VIP)/secretin/glucagon peptide family and has two biologically active isoforms—PACAP-27 and PACAP-38, with the latter being predominant in mammals [1]. PACAP acts through G-protein-coupled receptors: its specific receptor is PAC1, while VPAC1 and VPAC2 receptors bind PACAP and VIP with the same affinity. PACAP has a widespread distribution in the body, with the highest expression levels in the nervous system and endocrine glands, where it acts as a neurotransmitter, neuromodulator, and neurohormone [2,3,4,5,6]. In addition, PACAP and its receptors are widely expressed in peripheral organs [1,7,8,9,10], and the peptide plays different roles in numerous physiological processes in the cardiovascular, respiratory, urogenital, musculoskeletal, and digestive systems [11,12,13,14,15,16]. One of the established effects of the neuropeptide is its neurotrophic/neuroprotective action [17,18,19,20,21,22]. This has been proven in numerous neuronal insults and models of neurodegenerative diseases, such as spinal atrophy [23], amyotrophic lateral sclerosis [24], Alzheimer’s disease [25,26,27], stroke [28,29,30], Parkinson’s disease [20,31,32,33], Huntington chorea [34], and several types of retinal injuries [21,35,36,37]. 

Several reports have proven that PACAP is also protective in diabetes-related pathological conditions, such as diabetic nephropathy [38,39] and retinopathy [40,41,42,43,44,45,46]. It has been demonstrated that PACAP is protective in the inner, neuronal retinal layers in diabetic retinopathy and also acts on pigment epithelial cells in hyperglycemic conditions [47,48,49]. Vasculopathy is in the background of several diabetic complications. PACAP has been shown to ameliorate hyperglycemia-induced vascular dysfunction in isolated vessels [50]. Diabetic neuropathy is a common microvascular complication of diabetes, affecting around 50–70% of diabetic patients [51,52]. PACAP is involved in glucose metabolism and insulin secretion [53,54], in addition to protective effects exerted on the insulin-producing pancreatic beta cells [55,56]. More importantly, from a neuropathy point of view, PACAP influences Schwann cell functions [57,58], stimulates axonal growth [59,60], and promotes regeneration of peripheral nerves [61,62,63,64]. However, it is not known whether it has protective effects in diabetic neuropathy. Therefore, in the present study, we aimed at investigating the neuroprotective effects of PACAP in an experimental model of diabetic neuropathy in rats. 

The two major predictors of developing neuropathy are the duration of diabetes and the degree of metabolic instability [65]. Hyperglycemia results in excessive production of reactive oxygen species (ROS), overproduction of advanced glycation end products and glutamate, and decreased production of neuroprotective factors and hyperglycemia-activated signaling pathways, such as the polyol, hexosamine, and DAG-PKC pathways [65,66,67,68,69]. Signs and symptoms of diabetic neuropathy include loss of reflexes, dysesthesia, and paresthesia, along with neuropathic pain—hyperalgesia and allodynia [70]. Diabetic neuropathy is associated with alterations in the structure of peripheral nerves as well as in central structures of the pain processing pathway. However, the mechanism of neuropathic pain is still not understood, and it is an unmet medical need due to the ineffectiveness of the currently available therapy in a great proportion of cases. Previously, it has been shown that streptozotocin (STZ)-induced diabetes leads to alterations in the dorsal horn of the spinal cord and the mesencephalic periaqueductal grey matter (PAG) [71]. Among others, altered c-Fos and FosB expressions in these centers have been reported [72,73,74,75,76]. Therefore, in the present study, we also investigated the expression of FosB, a marker of chronic neuronal activation [77,78], in the above pain processing centers, in addition to the detailed morphological analysis of peripheral nerve fibers and functional assessment of pain sensation. 

## 2. Results

### 2.1. Blood Glucose Levels and Body Weight

**Figure 1:**
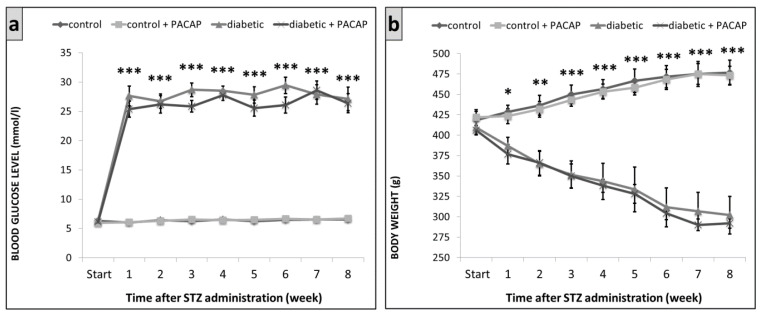
Change of blood glucose levels (**a**) and body weight (**b**) in control and diabetic groups. Data show mean ± SEM of *n* = 5/6 rats/group, * *p* < 0.05, ** *p* < 0.01, *** *p* < 0.001 vs. control groups.

Vehicle (saline) treated diabetic and PACAP-treated diabetic groups had a significant rise in blood sugar levels after the 7th day of the experiment. Blood glucose level was 6.6 ± 0.34 mmol/L in the vehicle-treated control and 6.7 ± 0.24 mmol/L in the PACAP-treated control group on week 8. These values were 27.1 ± 2.02 mmol/L in the vehicle-treated diabetic and 26.4 ± 1.65 mmol/L in the PACAP-treated diabetic group (Figure 1a). These data show that blood glucose levels were not significantly affected by PACAP treatment.

Vehicle-treated diabetic and PACAP-treated diabetic groups showed a significant decrease in their body weight from the second week. On week 8, we measured 477 ± 15 g in the vehicle-treated control, 473 ± 11 g in the PACAP-treated control groups, while only 302 ± 23 g in the diabetic and 292 ± 6 g in the PACAP-treated diabetic groups (Figure 1b). PACAP-38 treatment did not influence the body weight loss.

### 2.2. Functional Tests

#### 2.2.1. Randall–Selitto Test

**Figure 2:**
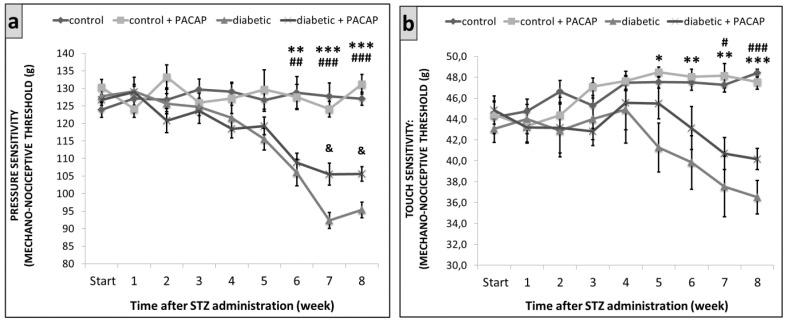
Diabetes-induced mechanical hyperalgesia in Wistar rats, the mechanical nociceptive threshold of pressure sensitivity by Randall-Selitto test (**a**) and touch sensitivity by dynamic plantar aesthesiometer (DPA) test (**b**). Data are presented as means ± SEM of 5/6 rats/group. * *p* < 0.05, ** *p* < 0.01, *** *p* < 0.001 vs. vehicle-treated diabetic group, ^#^ *p* < 0.05, ^##^ *p* < 0.01, ^###^ *p* < 0.001 vs. PACAP-treated diabetic group, ^&^ *p* < 0.05 vs. vehicle-treated diabetic group.

The Randal–Selitto test revealed an increased pressure sensitivity in the vehicle-treated diabetic and PACAP-treated diabetic groups after the 6th week of the experiment, compared to the vehicle-treated control and PACAP-treated control groups. Pressure sensitivity increased to a lower extent in the PACAP-treated diabetic group on the 7th and 8th weeks, compared to the vehicle-treated diabetic group (Figure 2a).

#### 2.2.2. Dynamic Plantar Aesthesiometer (DPA)

The DPA test showed an increased touch sensitivity in the vehicle-treated diabetic and PACAP-treated diabetic groups after the 5th week of the experiment, compared to the vehicle-treated control and PACAP-treated control groups. It was less prominent in the PACAP-treated diabetic group from the 5th week, compared to the vehicle-treated diabetic group, but the difference was not statistically significant (Figure 2b).

### 2.3. Immunohistochemistry

**Figure 3:**
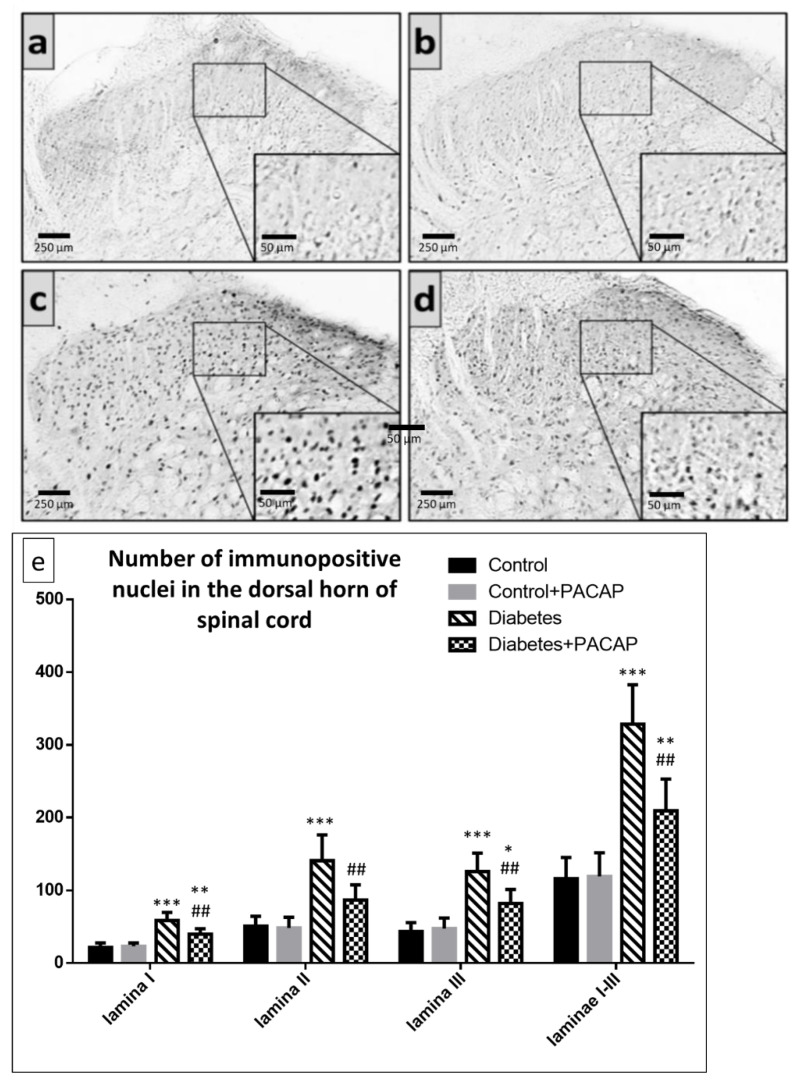
Representative images of FosB immunohistochemistry in the spinal dorsal horn of segments L4–L5 in (**a**) vehicle-treated control, (**b**) PACAP-treated control, (**c**) vehicle-treated diabetic, and (**d**) PACAP-treated diabetic groups. Histograms (**e**) show the number of FosB immunoreactive nuclei in laminae I-III of the spinal dorsal horn of segments L4–L5. Data are means ± SEM of *n* = 5/6 rats/group.* *p* < 0.05, ** *p* < 0.01, *** *p* < 0.001 vs. vehicle-treated control, ^##^ *p* < 0.01 vs. vehicle-treated diabetic group.

Our study showed that the number of FosB immunoreactive nuclei in laminae I-III of the spinal cord dorsal horn of segments L4–L5 was higher in the vehicle-treated diabetic and PACAP-treated diabetic groups, compared to the vehicle-treated control and PACAP-treated control groups. PACAP-treated diabetic animals had, however, significantly fewer FosB positive nuclei than vehicle-treated diabetic rats (Figure 3).

**Figure 4:**
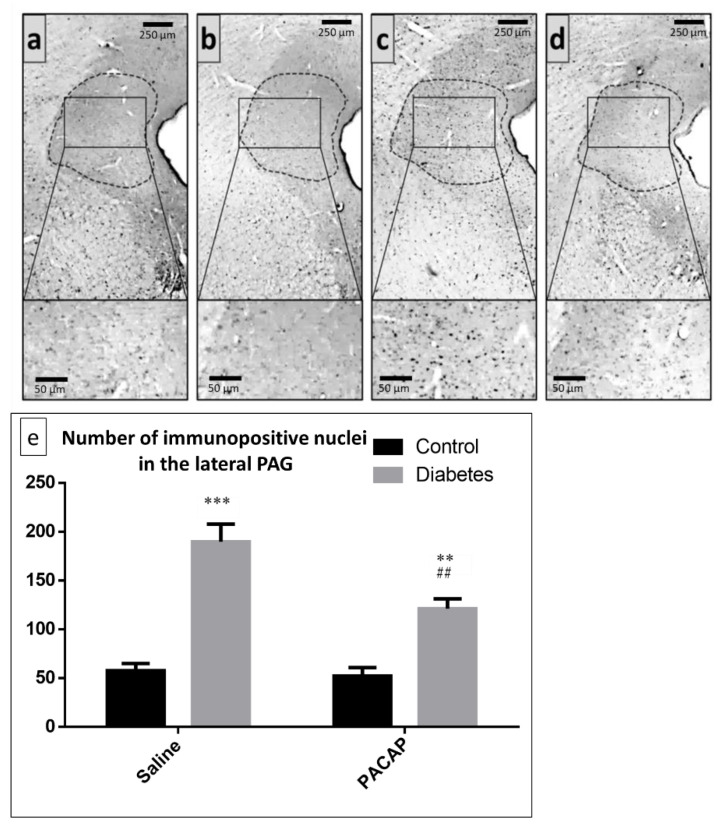
Representative images of FosB-positive nuclei in the lateral part of PAG (marked by dotted lines) (**a**) in vehicle-treated control, (**b**) PACAP-treated control, (**c**) vehicle-treated diabetic, and (**d**) PACAP-treated diabetic groups. Histogram (**e**) shows the number of FosB-positive/immunoreactive nuclei in the lateral part of PAG in vehicle-treated control, vehicle-treated diabetic, PACAP-treated control, and PACAP-treated diabetic groups. Data are means ± SEM of *n* = 5/6 rats/group. ** *p* < 0.01, *** *p* < 0.001 vs. vehicle-treated control, ^##^ *p* < 0.01 vs. vehicle-treated diabetic groups.

The number of FosB-positive nuclei in the lateral part of PAG was also investigated. We found that both vehicle- (saline) and PACAP-treated diabetic animals showed an elevated number of FosB immunoreactive nuclei in the lateral PAG, compared to the vehicle-treated control and PACAP-treated control groups. When comparing the PACAP-treated diabetic group to the vehicle-treated diabetic group, we found that PACAP was effective in significantly reducing the neuronal activity in the lateral PAG (Figure 4).

### 2.4. Electron Microscopy of the Sciatic Nerve 

**Figure 5:**
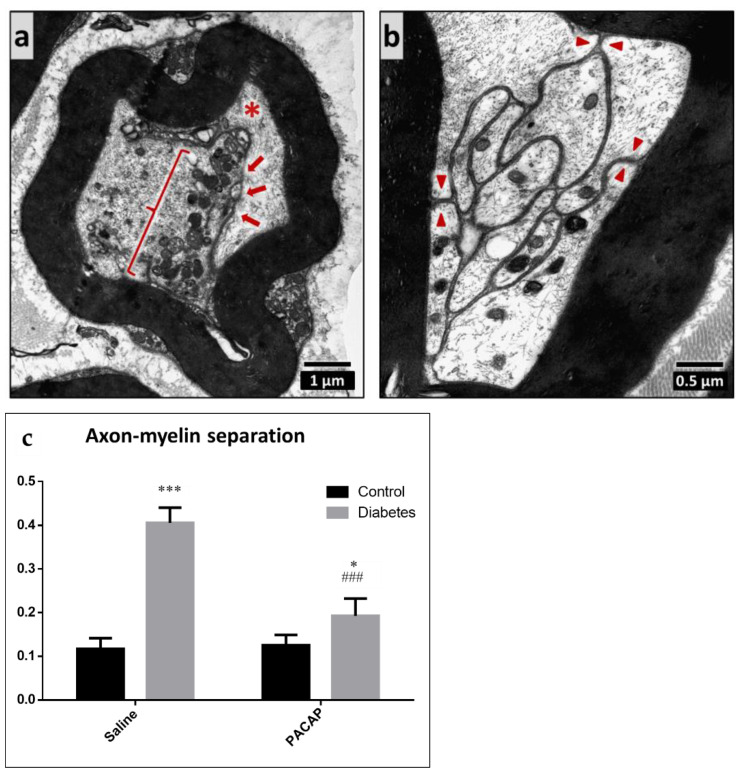
Representative electron microscopic images of axon–myelin separation in myelinated axons. The cell membrane of the Schwann cell is detached from the myelin sheath (arrows) and the axon is dislocated (star) (**a**). The separated cell membrane of the Schwann cell is visible (arrowheads, (**b**)). Histogram (**c**) shows the ratio of the axon–myelin separation in vehicle-treated control, vehicle-treated diabetic, PACAP-treated control, and PACAP-treated diabetic groups. Data are shown as means ± SEM of *n* = 5/6 rats/group. * *p* < 0.05, *** *p* < 0.001 vs. vehicle-treated control; ^###^ *p* < 0.001 vs. vehicle-treated diabetic group.

In vehicle-treated control and PACAP-treated control animals, normal peripheral nerve structure was found without any signs of myelin or axonal injury. PACAP treatment did not cause any changes under control (no diabetes) situations. However, the sciatic nerve of diabetic animals showed signs of neuropathy: axon–myelin separation, elevated average mitochondrial number in the axons, unmyelinated fiber atrophy, and basement membrane thickening. The percentage of the axon–myelin separation was significantly higher in the diabetic groups, compared to the control groups. However, it was significantly less prominent in the PACAP-treated diabetic group (Figure 5). 

**Figure 6:**
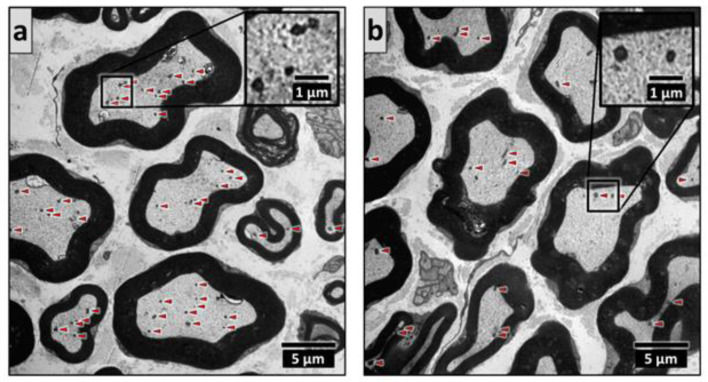

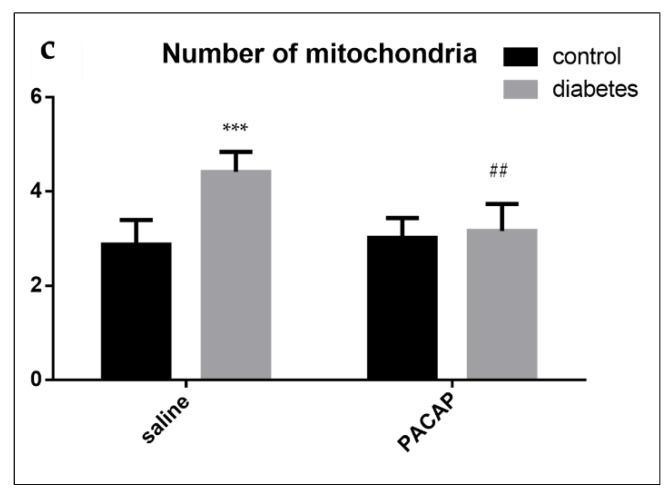
Representative electron microscopic images of diabetes-induced mitochondrial fission in vehicle-treated diabetic (**a**) and PACAP-treated diabetic (**b**) groups. Mitochondria are marked by arrowheads. Histogram (**c**) shows the average mitochondrial number in one myelinated axon in vehicle-treated control, vehicle-treated diabetic, PACAP-treated control, and PACAP-treated diabetic groups. Data are shown as means ± SEM, *n* = 5/6 rats/group. *** *p* < 0.001 vs. vehicle-treated control, ^##^ *p* < 0.01 vs. vehicle-treated diabetic groups.

A marked elevation in the mitochondrial number in the myelinated axons was found in the vehicle-treated diabetic group. This could not be observed in the PACAP-treated diabetic group; thus, PACAP successfully prevented the rise in mitochondrial number (Figure 6). 

**Figure 7:**
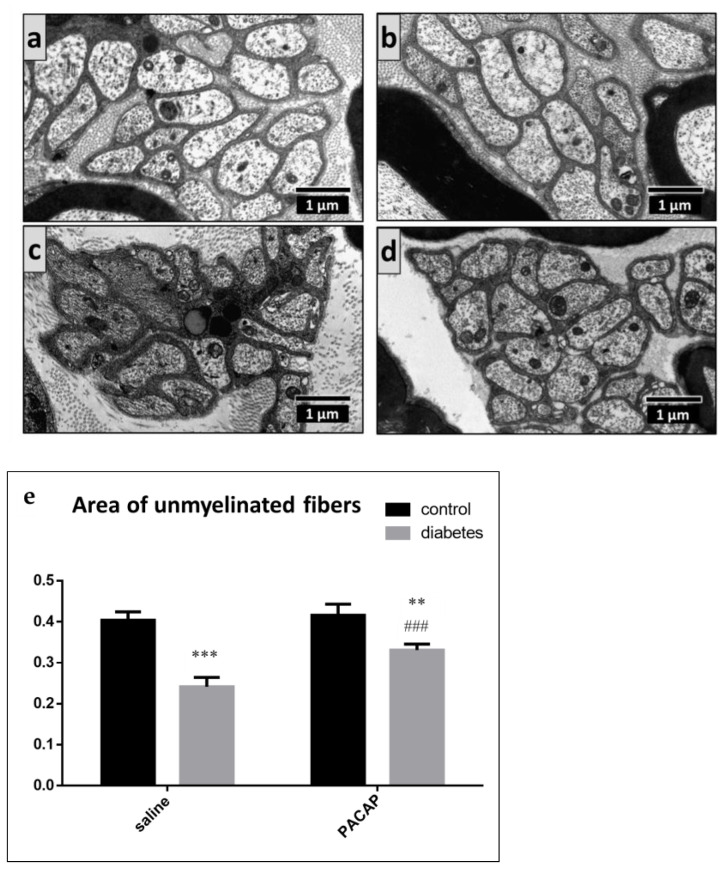
Representative electron microscopic images of unmyelinated fibers in vehicle-treated control (**a**), PACAP-treated control (**b**), vehicle-treated diabetic (**c**), and PACAP-treated diabetic (**d**) groups. Histogram (**e**) shows the area of the unmyelinated fibers (**e**) in vehicle-treated control, PACAP-treated control, vehicle-treated diabetic, and PACAP-treated diabetic groups. Data are represented as means ± SEM of *n* = 5/6 rats/group. ** *p* < 0.01, *** *p* < 0.001 vs. vehicle-treated control; ^###^ *p* < 0.05 vs. vehicle-treated diabetic groups.

We also found unmyelinated fiber atrophy, characterized by a decrease in the unmyelinated fiber area in the vehicle-treated diabetic and PACAP-treated diabetic groups, compared to the vehicle-treated control and PACAP-treated control groups. This decrease was significantly less severe in the PACAP-treated diabetic group. No difference was observed between the vehicle-treated control and PACAP-treated control groups (Figure 7). 

**Figure 8:**
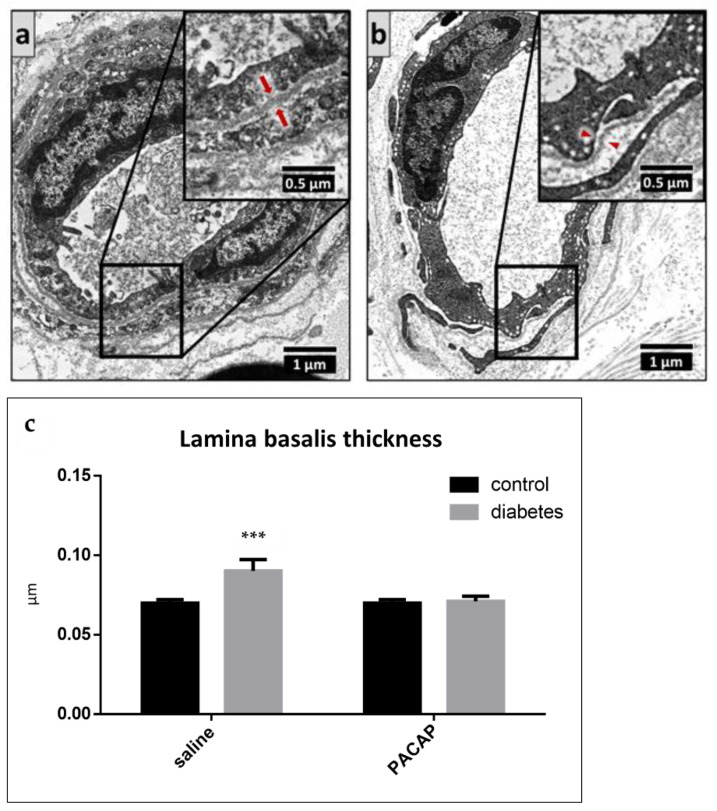
Representative electron microscopic images of the basement membrane of endoneurial capillaries in vehicle-treated diabetic ((**a**), arrows) and PACAP-treated diabetic ((**b**), arrowheads) groups. Histogram (**c**) shows the thickening of the basement membrane of the endoneurial capillaries in vehicle-treated control, PACAP-treated control, vehicle-treated diabetic, and PACAP-treated diabetic groups. Data are shown as means ± SEM of *n* = 5/6 rats/group, *** *p* < 0.001 vs. vehicle-treated control groups.

Electron microscopy also revealed thickening of the basement membrane in the endoneurial capillaries in the vehicle-treated diabetic group. PACAP-treated diabetic animals did not show any basement membrane thickening; there was no difference between the vehicle-treated control, PACAP-treated control, and PACAP-treated diabetic groups (Figure 8).

## 3. Discussion

In the present study, we demonstrated that in vivo PACAP treatment is protective in diabetic neuropathy. An 8-week PACAP-38 treatment effectively counteracted the functional and morphological changes observed in diabetic rats without altering the blood glucose levels or body weight. This observation is in accordance with our previous results studying the effects of PACAP treatment in diabetic nephropathy [38]. These data suggest that PACAP, in spite of its effects on glucose homeostasis and insulin secretion [53,54], is protective in our diabetic neuropathy model, not by acting directly on glucose levels, but most probably due to its neuro- and general cytoprotective effects [20,79]. Although, as outlined above, PACAP is known to stimulate insulin secretion, it did not alter blood glucose levels in this model of type I diabetes. Whether it affects insulin levels in this model or in a model of type II diabetes awaits further investigations. 

In the present study, we found that STZ-treated control diabetic rats displayed the morphological signs of diabetic neuropathy, i.e., axon–myelin separation, an increase in axonal mitochondria number, unmyelinated fiber atrophy, and basement membrane thickening of perineurial vessels. All these signs were attenuated by in vivo PACAP treatment. 

Previous studies have reported that axon–myelin separation is due to hyperglycemia [80], Na^+^ channel dysfunction [81], and glycogen accumulation in Schwann cells [82], which led to a hyperosmolar perineurial environment, causing axon–myelin separation and demyelination [83]. Our study showed that PACAP treatment markedly attenuated this axon–myelin separation. PACAP is known to be involved in myelin maturation and synthesis by inducing the expression of myelin markers [58,84], and it has a trophic and antiapoptotic effect on Schwann cells [57,85]. PACAP receptors are upregulated in peripheral nerve injury in the Schwann cells, and the peptide promotes myelin gene expression, inhibits the release of pro-inflammatory cytokines, and stimulates anti-inflammatory cytokines [86,87].

In experimental diabetic neuropathy, the increase in mitochondrial number has been reported in myelinated axons [80,88,89]. Presumably, hyperglycemia-induced oxidative stress stimulates mitochondrial fission, which leads to the overproduction of mitochondrial ROS resulting in small aberrant, more electron-dense mitochondria with a reduced respiratory capacity [88]. It has been suggested that the inhibition of mitochondrial fission would relieve the ROS-induced oxidative stress [90]. The attenuated response in PACAP-treated animals might be due to the ability of PACAP to decrease oxidative metabolite levels, increase antioxidant potential [91], and stimulate antioxidant enzymes, such as peroxiredoxin [92], heme oxygenase-1 [93], superoxide-dismutase [94], and glutathione [39,95].

Unmyelinated fiber atrophy was found in STZ-induced diabetic rats, with a reduced cross-sectional area of the axons, similar to other reports [96]. This could also be attenuated by PACAP treatment. The protective effects of PACAP in nerve degeneration have been confirmed by dozens of studies [97,98]. Among others, PACAP promotes cell survival and neurite outgrowth [62,99], enhances axonal sprouting [100], and stimulates neuronal differentiation during development and regeneration [64,101,102,103,104]. In peripheral nerve injuries, PACAP has been shown to be upregulated and to promote regeneration partly through stimulation of other growth factors, such as glial cell line-derived neurotrophic factor [97,105,106,107]. Given the importance of endogenous PACAP in nerve regeneration, not surprisingly, mice lacking endogenous PACAP show a slower axonal regeneration with an increased pro-inflammatory environment [61]. These authors suggested that endogenous PACAP is involved in the controlled immune response that is necessary for proper nerve regeneration after injury [61]. This action of PACAP has been recently supported by human data: transcriptional profiling of the skin from patients with carpal tunnel syndrome revealed that the gene encoding PACAP was the most strongly upregulated gene and its expression was associated with recovery of intraepidermal nerve fibers [62]. 

Diabetes is also associated with the thickening of the basement membrane of the vasa nervosum as a consequence of the increased expression and decreased breakdown of collagen IV [108,109,110]. We found that diabetes resulted in the thickening of the basement membrane, attenuated by PACAP treatment. Similar to our present results, PACAP was found to attenuate basement membrane thickening in diabetic nephropathy [39]. Similar vascular protective effects have been observed in murine endothelial cells exposed to glucose: PACAP elicited an antiproliferative effect under chronic hyperglycemic conditions [111]. PACAP has also been demonstrated to protect endothelial cells against oxidative stress [112]. The protective effects on vessels are reflected in morphological signs, but PACAP has also been shown to reduce hyperglycemia-induced vascular dysfunction [50]. In that study, PACAP restored the disturbed relaxation of the vessels to an extent comparable to superoxide dismutase without direct scavenging of ROS. The elevated levels of fibroblast growth factor basic, matrix metalloproteinase 9, and nephroblastoma associated with endothelial dysfunction could be reduced by PACAP administration [50]. The model used in our study mimics type I diabetes, as streptozotocin leads to toxic degeneration of the insulin-producing beta cells. Based on these observations, however, it is plausible that PACAP could also be protective in neuropathies observed in type II diabetes, as the main mechanism of neuropathic induction is not directly related to the model itself but more to the increased glucose levels. However, in order to prove this point, further experiments are required. 

The observed morphological signs of the protective effects of PACAP were also reflected in functional improvement in our study. Mechanical hyperalgesia is present in early diabetic neuropathy [113]. In our study, the intraperitoneally administered PACAP significantly attenuated the enhancement of pressure sensitivity by measuring mechanical hyperalgesia. In addition to the protective effects on nerve fibers, the anti-nociceptive and anti-hyperalgesic effects of PACAP might also be due to the decreased release of the pro-nociceptive neuropeptides [114]. In our present study, we also investigated the activation of pain-processing central structures. The expression of the acute neuronal activity marker c-Fos has been shown to be increased in the STZ diabetic model in the PAG and dorsal horn of the spinal cord [71]. Previous studies have found that FosB expression is significantly elevated in rats in the case of chronic pain and stress but not acute pain [115,116]. The dorsal horn of segment L4 of the spinal cord corresponds to the primary nociceptive afferent regions of the rat’s hind paw [117], while the lateral PAG is an important center of the descending anti-nociceptive system [118]. Here, we described chronic neuronal activity (i.e., FosB expression) in the spinal dorsal horn of segments L4–L5 and in the lateral part of PAG in our STZ diabetes model and found that PACAP treatment effectively prevented FosB activation in these centers. Our earlier findings in mice lacking endogenous PACAP support these findings, as PACAP knockout mice showed increased basal neuronal activity (i.e., c-Fos) in the lateral PAG [119]. The importance of endogenous PACAP in pain-processing centers has been highlighted also by several other studies [120,121,122,123]. 

In conclusion, here we show, for the first time, that PACAP treatment can attenuate or moderate the pathological changes of diabetic neuropathy, suggesting that PACAP could have therapeutic potential in diabetic neuropathy. 

## 4. Materials and Methods

### 4.1. Animals

The experiment was carried out using adult male Wistar rats (*n* = 22) weighing 360–420 g. The experimental animals were housed under light/dark cycles of 12:12 h and received normal rat chow and drinking water ad libitum. Rats were randomly divided into four groups: (1) vehicle (saline)-treated control (non-diabetic) (*n* = 5); (2) PACAP-treated control (non-diabetic) (*n* = 5); (3) vehicle-treated diabetic (*n* = 5), and (4) PACAP-treated diabetic (*n* = 5) groups (in the figures, these groups are referred to as control, control + PACAP, diabetic, diabetic + PACAP groups, respectively). Diabetes was induced by a single dose of 65 mg/kg intravenous streptozotocin injection (Sigma, Budapest, Hungary). PACAP (20 µg PACAP1-38/100 μL saline solution) was injected intraperitoneally every second day for eight weeks, starting simultaneously with the streptozotocin injection to PACAP-treated control and PACAP-treated diabetic groups. The dose of PACAP was based on previous observations where this dose was effective in a rat model of diabetic nephropathy [38,39]. Vehicle-treated control and vehicle-treated diabetic groups received 100 μL saline intraperitoneally. Body weight and blood glucose levels (Accu-Check Active, Roche, Budapest, Hungary) were measured weekly, rats with glucose levels higher than 11 mmol/L were considered diabetic. Experimental procedures were carried out in accordance with approved protocols (University of Pecs; BA02/2000-24/2011). We performed in vivo behavioral tests on all experimental animals of the four groups. Following 8 weeks of survival, animals were processed for histological analysis.

### 4.2. Functional Tests

#### 4.2.1. Mechanical Nociceptive Threshold—Randall–Selitto Test

The pressure sensitivity of the hind paw was measured by the Randall–Selitto test using Ugo Basile Analgesia Meter on a weekly basis. During the Randall–Selitto test, a continuously increasing pressure—at a maximum of 250 g—was applied to the hind paw of the rats. The increasing pressure caused the withdrawal of the paw, which was considered as the mechanical nociceptive threshold in grams [124,125]. Three measurements were made on both left and right hind paws, and the average of the assessments was taken. A decreased mechanical nociceptive threshold in this test can be considered as hyperalgesia [113]. 

#### 4.2.2. Mechanical Nociceptive Threshold—Dynamic Plantar Aesthesiometer Test (DPA)

Touch sensitivity on the plantar surface of the hind paws was determined by a dynamic plantar aesthesiometer (DPA) (Ugo Basile, Gemonio, Italy) on a weekly basis. During the DPA test, a continuously increasing force—at a maximum of 50 g in 10 s—was applied to the hind paw by the elevation of a blunt-end needle and the aesthesiometer automatically detected the latency time and force (in grams) at the time of paw withdrawal. The decreased mechanical nociceptive threshold in the DPA test is a sign of allodynia since this mechanical stimulus is not painful to the rats [126].

### 4.3. Histology

#### 4.3.1. Tissue Collection and Preparation for Histology

Animals were anesthetized with an overdose of isoflurane (Forane, Abbott Hungary, Budapest, Hungary) on week 8. Rats were transcardially perfused with 25 mL of phosphate-buffered saline (PBS), followed by 300 mL of 4% paraformaldehyde solution in Millonig buffer for 20 min. The brain and spinal cord were dissected and then placed into the same fixative solution for post-fixation for 72 h at 4 °C. The sciatic nerve was also removed from all animals and further processed for electron microscopy.

A tissue block containing the midbrain was isolated from the brains by cutting at the frontal planes of the posterior border of the median eminence and the transverse fissure. A tissue block of the L4-L5 spinal cord segments was also dissected. Blocks were sectioned by a Leica VT S 1000 (Leica, Wetzlar, Germany) vibratome. Five series of 30 µm coronal sections, interspaced by 120 µm, were collected in anti-freeze solution and stored at −20 °C until further use.

#### 4.3.2. Immunohistochemistry for FosB

Free-floating labeling for the chronic neuronal activation marker FosB was performed on a series of the midbrain and spinal cord sections, as published earlier [127]. Briefly, sections were permeabilized with 0.5% Triton X-100 solution. Normal goat serum (2%, NGS, Jackson Immunoresearch, Europe Ltd., Suffolk, UK) was used to reduce non-specific binding. Subsequently, sections were treated with rabbit anti-FosB antibodies (1:500, Santa Cruz, sc-48 Santa Cruz Biotechnology Inc., Santa Cruz, CA, USA) in PBS with 2% NGS overnight. After washes, sections were treated with biotinylated goat anti-rabbit serum and avidin–biotin complex (Vectastain ABC Elite Kit Vector Lbs., Burlingame, CA, USA) according to the supplier’s protocol. The labeling was developed in Tris buffer (pH 7.4) with 0.02% 3, 3′ diamino-benzidine (DAB) (Sigma) and 0.00003 *v*/*v*% H_2_O_2_. The reaction was carried out under visual control and was stopped after 7 min with PBS. Then, preparations were washed and mounted on gelatin-covered slides. After drying, sections were dehydrated with ethanol solutions (50%, 70%, 96%, absolute, absolute 5 min, respectively), cleared by xylene (2 × 20 min), and coverslipped using Depex (Fluka, Heidelberg, Germany). Specificity of the FosB antiserum (Santa Cruz, sc-48) was characterized earlier [128,129]. Western blot studies also support the specificity (http://datasheets.scbt.com/sc-48.pdf, 2017). Preabsorption experiment in the rat revealed that the blocking peptide (sc-48-P, Santa Cruz) prevented the immunolabeling. In line with this, omission and/or replacement of the primary or secondary antibodies by non-immune sera abolished the signal in all tests (images not shown).

#### 4.3.3. Digital Imaging and Morphometry at Light Microscopic Level

The DAB-labeled FosB immunohistochemistry was studied and digitalized by a Nikon Microphot FXA microscope with a Spot RT camera (Nikon, Tokyo, Japan). For each animal, five serial sections were photographed. The number of FosB positive nuclei was determined using non-edited digital images by manual cell counting. The whole cross-sectional surface areas of the lateral PAG, as well as the dorsal horn laminae I, II, and III, individually were measured, and summed (laminae I + II + II) at L4–L5 spinal segments were evaluated, as marked in Figure 3 and Figure 4. Cell counting was carried out by a skilled neurophysiologist who was not informed about the identity of preparations. For documentation and publication purposes, the micro-photos were grey-scaled and contrasted using Adobe Photoshop 7.0.1 software.

#### 4.3.4. Electron Microscopy

Sciatic nerve samples were placed in fixing solution (2.5% glutaraldehyde + 2% formalin + 0.1 M PBS) immediately after dissection in +4 °C for 24 h. A post-fixation procedure was performed with 1% osmium tetroxide. After dehydration in ascending alcohol (50%–70%–90%–96%) and subsequent transfer to propylene oxide, samples were embedded in Araldite resin. Semithin sections (0.5 µm) were cut by ultramicrotome (Leica Ultracut R), stained with 1% toluidine blue (Sigma), and examined with a Nikon Eclipse 80i microscope. Ultrathin sections were prepared from the area of interest and were contrasted by 2.5% uranyl–acetic acid and lead citrate. Slides were examined using a JEM-1200 EX-II electron microscope. The following parameters were analyzed: percentage of the axon-myelin separation, mitochondrial number in myelinated axons, area of the unmyelinated fibers, and thickness of the basement membrane.

### 4.4. Statistical Analysis

Statistical analysis was performed in GraphPad Prism 6.01 software. Two-way analysis of variance (ANOVA) was used to detect significant differences between groups. Multiple comparisons were performed by Tukey’s test. Data are presented as means ± S.E.M (standard error of the mean). Differences were considered statistically significant when *p* < 0.05.

## Data Availability

The data presented in this study are available in the article, there is no supplementary data.
